# Retraction: Jiang et al. Formation of Proto-Kranz in C3 Rice Induced by Spike-Stalk Injection Method. *Int. J. Mol. Sci*. 2021, *22*, 4305

**DOI:** 10.3390/ijms222111930

**Published:** 2021-11-03

**Authors:** International Journal of Molecular Sciences Editorial Office

**Affiliations:** MDPI, St. Alban-Anlage 66, 4052 Basel, Switzerland; ijms@mdpi.com

The journal and the authors retract the article “Formation of Proto-Kranz in C3 Rice Induced by Spike-Stalk Injection Method” [1] cited above.

Following publication, concerns were brought to the attention of the editors regarding experimental results from [1] that were found to be unreliable, namely: (1) Several sorghum genes were integrated into the rice genome; (2) Sorghum PPDK and PEPCK genes were expressed in the sorghum–rice (SR) plant; (3) Integration of sorghum DNA caused proto-Kranz anatomy. This has brought about uncertainty regarding the scientific conclusions. 

In accordance with our ethics procedures, an investigation was conducted. The authors have not been able to provide a satisfactory explanation for these irregularities and the journal’s Editors-in-Chief no longer has confidence in the conclusions of the paper. Therefore, to ensure the addition of only high-quality scientific works to the field of scholarly publication, this paper [1] is retracted and shall be marked accordingly. The authors agree with this retraction.

We apologize to our readership that this went undetected until now.
